# Clinical and pathological findings of IgA nephropathy following SARS-CoV-2 infection

**DOI:** 10.1007/s10238-023-01271-2

**Published:** 2024-02-24

**Authors:** Hongfen Li, Zhao Li, Zhanfei Wu, Fanghao Wang, Yue Xing, Youxia Liu, Junya Jia, Tiekun Yan

**Affiliations:** 1 Department of Nephrology, Tianjin Medical University General Hospital, No. 154, Anshan Road, Heping District, Tianjin, People’s Republic of China; 2 Department of Clinical Discipline of Chinese and Western Integrative Medicine, Shuanghuan Cun Street Community Health Services Center, Tianjin, People’s Republic of China

**Keywords:** IgA nephropathy, SARS-CoV-2, Clinical features, Pathological characteristics

## Abstract

The features of IgA nephropathy (IgAN) after SARS-CoV-2 infection have not been well characterized. In this study, we compared the clinical and pathological characteristics of patients with IgAN who had experienced SARS-CoV-2 infection to those who had not. We conducted a retrospective study that enrolled 38 patients with biopsy-proven IgAN following SARS-CoV-2 infection with 4 months (post-SARS-CoV-2 infection group) and 1154 patients with IgAN prior to the pandemic (pre-SARS-CoV-2 infection group). Among the SARS-CoV-2 group cases, 61% were females. The average duration from SARS-CoV-2 infection to renal biopsy was 78.6 days. Prior to SARS-CoV-2 infection, the patients had different presentations of nephropathy. One patient had isolated hematuria, two had isolated proteinuria, twenty presented with both hematuria and proteinuria, and one patient had elevated serum creatinine. Additionally, there were eight cases with uncertain nephropathy history, and six cases did not have a history of nephropathy. Following SARS-CoV-2 infection, five patients experienced gross hematuria, one case exhibited creatinine elevation, and five cases showed an increase in proteinuria. The group of patients infected with SARS-CoV-2 after the COVID-19 pandemic exhibited older age, higher hypertension ratio and lower eGFR values compared to the pre-SARS-CoV-2 infection group. As for pathological parameters, a higher proportion of patients in the post-SARS-CoV-2 infection group exhibited a higher percentage of sclerotic glomeruli and glomerular ischemic sclerosis. There were no significant differences observed between the two groups in terms of therapy involving steroids, immunosuppressants, or RAS inhibitors. IgA nephropathy patients who were infected with SARS-CoV-2 were generally older and experienced more severe kidney damage compared to those without SARS-CoV-2 infection.

## Introduction

The COVID-19 pandemic has had a profound impact on global health, with the emergence of new variants of the SARS-CoV-2 virus posing ongoing challenges. While severe respiratory infections have been the prominent clinical manifestation of COVID-19, it is now evident that the virus can affect other organs beyond the respiratory system [1].

Increasing evidence suggests that SARS-CoV-2 virus can cause kidney damage and gastrointestinal symptoms in infected individuals. Kidney damage, including acute kidney injury and proteinuria, has been observed and has been associated with higher mortality rates in severe cases [2]. To date, several types of glomerular diseases have been identified in association with COVID-19, including collapsing glomerulopathy, lupus nephritis, IgA nephropathy (IgAN), and acute kidney failure [3]. IgAN is a common primary glomerular disease worldwide and has been previously associated with respiratory or intestinal infections [4]. However, it is still unclear whether the manifestation of IgAN is influenced by SARS-CoV-2 infection. The specific impact of SARS-CoV-2 on the development, severity or progression of IgAN is not well understood. Currently, there is limited available information regarding the clinical and pathological characteristics of patients with IgAN who have also contracted SARS-CoV-2. Given the limited number of case reports and studies investigating the relationship between SARS-CoV-2 infection and IgAN, further research is needed to determine any potential association and understand the underlying mechanisms.

The aim of this study was to assess the clinical and pathological characteristics of IgAN patients who with a history of SARS-CoV-2 infection. By conducting this study, the researchers sought to gather information and insights into how the presence of SARS-CoV-2 infection may influence the features of IgAN.

## Methods

### Study design and population

**Fig. 1:**
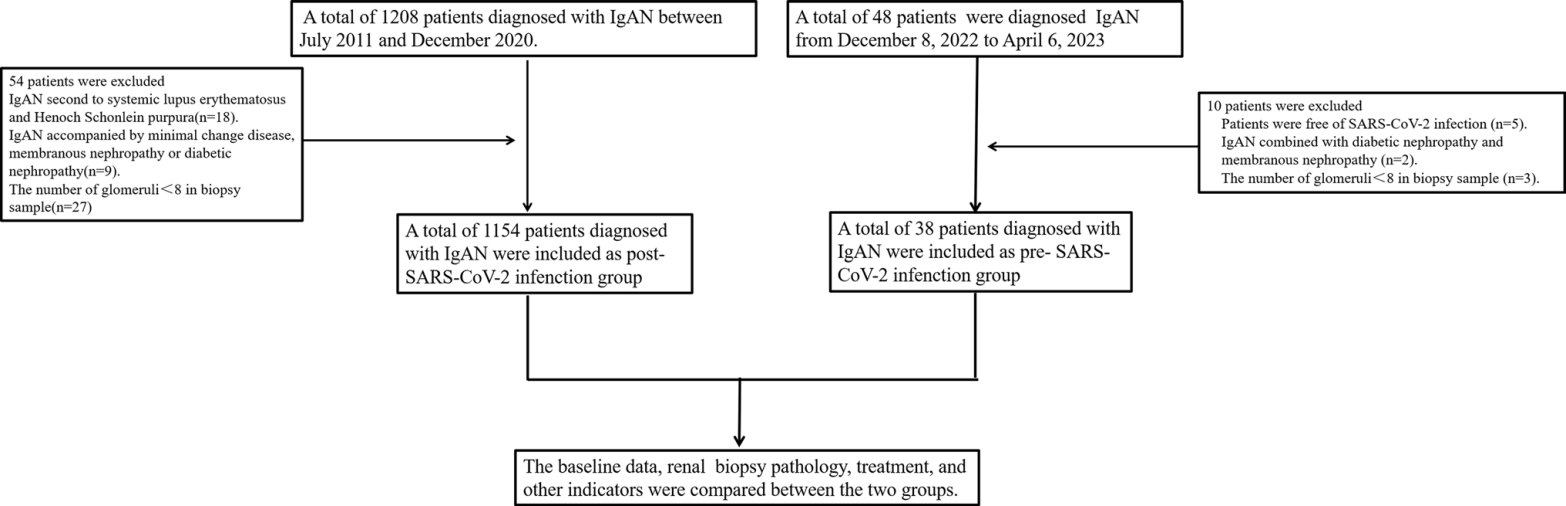
Flowchart of the recruitment process

This retrospective study involved a group of patients with IgAN at Tianjin Medical University General Hospital. A total of 1154 patients diagnosed with IgAN through renal biopsy, which included at least 8 glomeruli in our cohorts between July 2011 and December 2020, were analyzed. Patients with secondary IgAN, such as those with systemic lupus erythematosus and Henoch Schonlein purpura, were excluded from the study. Additionally, patients with IgAN accompanied by minimal change disease, membranous nephropathy or diabetic nephropathy were also excluded (Fig. 1).

From December 8, 2022, to April 6, 2023, a total of 48 patients were diagnosed IgAN in our hospital. These patients represented approximately 22.5% of the total number of patients who underwent renal biopsies during the specified time frame. Among them, five patients were found to be free of SARS-CoV-2 infection. Additionally, one patient had a concurrent diagnosis of diabetic nephropathy, one patient had a concurrent diagnosis of membranoproliferative glomerulonephritis, and three patients with less than eight glomeruli observed during renal biopsy were excluded from the study (Fig. 1).

This study was approved by the institutional ethics committee of Tianjin Medical University General Hospital, and all patients provided written informed consent.

### Clinical data collection

Demographic characteristics and clinical data including age, gender, hypertension ratio, diabetic ratio, systolic blood pressure (SBP), hemoglobin (Hb), erythrocyte sedimentation rate (ESR), blood platelet (PLT), prothrombin time (PT), activated partial thromboplastin time (APTT), fibrinogen (FIB), D-dimer, serum albumin (Alb), serum globulin (GLO), serum creatinine (Scr), estimated glomerular filtration rate (eGFR), serum uric acid (UA), proteinuria, Complement C3, Complement C4, urinary red blood cell, total cholesterol (TC), triglyceride (TG), low density lipoprotein (LDL), serum immunoglobulin A (IgA) and serum immunoglobuling G (IgG) were collected at the time of renal biopsy.

### Definitions

Individuals with a blood pressure of  ≥ 140/90 mmHg are considered hypertension [5].

The eGFR was calculated using the Chronic Kidney Epidemiology Collaboration (CKD-EPI) equation, which is a tool commonly used to assess kidney function in patients with CKD [6]. The histological lesions were categorized based on the Oxford classification scores (MEST-C, M: mesangial hypercellularity; E: endocapillary hypercellularity; S: segmental glomerulosclerosis; T: tubular atrophy/interstitial fibrosis, and C: crescent) [7]. The presence of more than 50% crescent in the glomerular cystic cavity was considered a larger crescent.

A renin-angiotensin system inhibitor (RASI) refers to the use of drugs such as angiotensin-converting enzyme inhibitors (ACEIs) and/or angiotensin receptor blockers (ARBs) after a biopsy. Immunosuppressive therapy was defined as treatment with cyclophosphamide, cyclosporine, or mycophenolate mofetil, following a kidney biopsy.

### Statistical analysis

Normally distributed continuous variables were compared using Student’s t test and expressed as mean ± standard deviation (SD). Non-normally distributed continuous data were compared using the Mann–Whitney U test and presented as medians and interquartile ranges. Dichotomous data were presented as both numerical values and percentages, and statistical comparison was performed using the χ^2^ test. The statistical analysis, conducted using SPSS 25.0 software, showed *P* < 0.05 (two-tailed) was considered significant.

## Results

### Baseline characteristics of IgAN patients following COVID-19 infection

A total of 38 patients who had contracted COVID-19 were included in this study.

Table 1 provides an overview of the demographic and clinical characteristics of these patients. The mean age of patients in the post-SARS-CoV-2 infection group was 45.89 years and the mean SBP was 135 mmHg. Among the COVID-19 cases included in the study, the majority (61%, n = 23) were female. Regarding the timing of renal biopsy, the mean duration from SARS-CoV-2 infection to the biopsy procedure was 78.6 days. Prior to SARS-CoV-2 infection, the patients had different presentations of nephropathy. One patient had isolated hematuria, two had isolated proteinuria, twenty presented with both hematuria and proteinuria, and one patient had elevated serum creatinine. Additionally, there were eight cases with uncertain nephropathy history, and six cases did not have a history of nephropathy. Following COVID-19 infection, five patients experienced gross hematuria, one case exhibited creatinine elevation, and five cases showed an increase in proteinuria (Table 1).

**Table 1:** Baseline characteristics of IgAN patients following SARS-CoV-2 infection

Characteristics	Mean ± SD, or n
Age (years)	45.89 ± 14.97
Gender (M/F)	15/23
SBP (mm Hg)	135 ± 17
Time to renal biopsy after SARS-CoV-2 infection (d)	78.6 ± 29.4
Isolated hematuria before SARS-CoV-2 infection (n)	1
Isolated proteinuria before SARS-CoV-2 infection (n)	2
Proteinuria and elevated serum creatinine before COVID-19 infection (n)	1
Hematuria and proteinuria before SARS-CoV-2 infection (n)	20
Uncertain nephropathy history before SARS-CoV-2 infection (n)	8
Denial of nephropathy history before SARS-CoV-2 infection (n)	6
Gross hematuria after SARS-CoV-2 infection (n)	5
Creatinine elevation after SARS-CoV-2 infection (n)	1
Proteinuria elevation after SARS-CoV-2 infection (n)	5

*y* years, *M* man, *F* femal, *SBP* systolic blood pressure, *d* days, *eGFR* glomerular filtration rate

### Clinical and pathological characteristics of IgAN patients in the post-COVID-19 and pre-COVID-19 infection groups

We compared the clinical and pathological characteristics of IgAN patients who had contracted COVID-19 to those who had not. As shown in Table 2, the group of patients infected with SARS-CoV-2 after the COVID-19 pandemic exhibited older age (45.89 ± 14.98 vs. 38.17 ± 12.53 years, *P* < 0.001), higher hypertension ratio (65.8% vs. 41.9%, *P* = 0.03) and lower eGFR values (77.36 ± 25.2 ml/min vs. 92.58 ± 31.31 ml/min, *P* = 0.03) than those in the pre-SARS-CoV-2 infection group. Although there was no statistically significant difference in the incidence of diabetes between the two groups, the post-SARS-CoV-2 infection group had a higher prevalence of diabetes (13.2% vs. 5.7%, *P* = 0.057). Based on the pathological parameters, the post-SARS-CoV-2 infection group showed a higher proportion of sclerotic glomeruli [13.2% (14.94%, 57.54%) vs. 11.54% (0.0, 28%), *P* < 0.001] and glomerular ischemic sclerosis [18.61% (6.67%, 31.41%) vs. 5.26% (0.0, 14.29%), *P* < 0.001] compared to the pre-SARS-CoV-2 infection group. Though there was no statistically significant difference in the tubular atrophy/interstitial fibrosis (T1/2), the post-COVID-19 infection group had a higher proportion of T1/2 lesions (73.7% vs. 60.6%, *P* = 0.057) compared to the pre-SARS-CoV-2 infection group (Table 3). There was no significant difference in gender, SBP, ESR, PLT, PT, APTT, FIB, D-Dimer, ALB, GLO, urinary protein, serum creatinine, uric acid, urinary red blood cell count, IgA, IgG, complement C3, C4, TC, TG, LDL between the two groups (Table 2). There were no differences observed between the two groups in terms of other pathological parameters, including the percentage of M1, E1, S1, C1/C2, sclerotic glomeruli, glomerular globally sclerotic, glomerular segmental sclerosis, crescents, large crescents, and fibrinoid necrosis (Table 3). No significant differences were observed between the two groups in terms of therapy involving steroids, immunosuppressants, or RAS inhibitors (Table 3).

**Table 2:** Comparison of baseline characteristics between IgAN patients post-COVID-19 and pre-COVID-19 infection

Variables	IgAN post-COVID-19 infenction group (n = 38)	IgAN pre-COVID-19 infection group (n = 1154)	*P* Value
Age (ys)	45.89 ± 14.98	38.17 ± 12.53	< 0.001
Gender (M/F)	15/23	536/618	0.396
Hypertension n (%)	25 (65.8)	484 (41.9)	0.003
Diabetics n (%)	5 (13.2)	66 (5.7)	0.057
SBP (mm Hg)	134.79 ± 16.72	131.04 ± 18.88	0.227
HB (g/l)	127.1 ± 17.8	130.4 ± 19.8	0.312
ESR (mm/h)	26.27 ± 12.73	22.15 ± 13.2	0.12
PLT (10^9/l)	260.21 ± 60.44	248.93 ± 64.98	0.292
PT (sec)	10.68 ± 1.69	11.21 ± 4.38	0.456
APTT (sec)	30.52 ± 2.32	29.06 ± 4.82	0.064
FIB (g/L)	3.40 ± 0.58	3.23 ± 1.59	0.524
D-Dimer (ng/ml, median, IQR)	268.0 (182.4, 444.5)	318.5 (192.0, 594.0)	0.932
Alb (g/l)	35.68 ± 4.55	37.33 ± 5.71	0.079
GLO (g/l)	27.97 ± 3.76	27.4 ± 4.46	0.430
Scr (umol/l)	94.26 ± 35.4	90.39 ± 52.43	0.651
eGFR (ml/min.1.73m^2^)	77.36 ± 25.2	92.58 ± 31.31	0.003
Proteinuria (g/24 h, median, IQR)	1120 (671, 2028)	1142 (590, 2263)	0.756
Uric acid (umol/L)	378.97 ± 85.43	366.93 ± 105.26	0.486
C3 (mg/dl)	88.20 ± 17.65	93.04 ± 22.62	0.204
C4 (mg/dl)	23.52 ± 6.37	23.14 ± 8.94	0.889
Urine RBC (/ul,median,IQR)	130.4 (32.7, 239.8)	134.2(31.5, 297.4)	0.835
TC (mmol/l)	5.16 ± 1.4	5.0 ± 1.5	0.522
TG (mmol/l)	2.43 ± 1.94	1.88 ± 1.38	0.06
LDL (mmol/l)	3.22 ± 0.96	3.0 ± 1.22	0.274
Total IgA (mg/ml)	343.22 ± 102.7	319.96 ± 130.92	0.292
Total IgG (mg/ml)	1134.25 ± 246.54	1054.01 ± 286.73	0.098

*SBP* systolic pressure, *HB* haemoglobin, *ESR* erythrocyte sedimentation rate, *PLT* blood platelet, *PT* prothrombin time, *APTT* activated partial thromboplastin time, *FIB* fibrinogen, *Scr* serum creatinine, *eGFR* estimated glomerular filtration rate, *Alb* albumin, *TC* total cholesterol, *TG* triglyceride, *LDL* low density lipoprotein, *C3* Complement C3, *C4* Complement C4, *Urine RBC* urinary red blood cell

**Table 3:** Comparison of renal perforation pathology and therapy in patients with IgAN after and before COVID-19 infection

Variables	IgAN post-COVID-19 infenction group (n = 38)	IgAN pre-COVID-19 infection group (n = 1154)	*P* Value
Pathological parameters (%)			
M (M0/M1)	1 (2.6) /37 (97.4)	20 (1.7) /1134 (98.3)	0.679
E (E0/E1)	23 (60.5) /15 (39.5)	732 (63.4) /422 (36.6)	0.715
S (S0/S1)	14 (36.8) /24 (63.2)	411 (35.6) /743 (64.4)	0.877
T (T0/T1/T2)	10 (26.3) /23 (60.5) /5 (13.2)	455 (39.4) /474 (41.1) /225 (19.5)	0.057
C (C0/C1/C2)	12 (31.6) /22 (59.7) /4 (8.7)	413 (35.8) /585 (50.7) /156 (13.5)	0.669
Sclerotic glomeruli (median, IQR, %)	31.39 (14.94, 57.54)	11.54 (0.0, 28)	< 0.001
Globally sclerotic (median, IQR, %)	0.0 (0.0, 7.69)	0.0 (0.0, 5.56)	0.356
Glomerular segmental sclerosis (median, IQR, %)	0.0 (0.0, 0.56)	0.0 (0.0, 8.27)	0.078
Glomerular ischemic sclerosis (median, IQR, %)	18.61 (6.67, 31.41)	5.26 (0.0, 14.29)	< 0.001
Crescents (median, IQR, %)	6.27 (0, 12.78)	7.14 (0.0, 15.79)	0.589
Large crescents (median, IQR, %)	0.0 (0.0, 0.0)	0.0 (0.0 ~ 3.05)	0.276
Fibrinoid necrosis (median, IQR, %)	0.0 (0.0, 5.72)	0.0 (0.0, 4.0)	0.194
Treatment			
Steroids n (%)	22 (57.9)	654 (56.7)	0.881
Immunosuppressors n (%)	31 (81.6)	781 (67.7)	0.07
RAS inhibitors n (%)	14 (36.8)	464 (40.2)	0.677

*M* mesangial hypercellularity, *E* endocapillary hypercellularity, *S* segmental glomerulosclerosis, *T* tubular atrophy/interstitial fibrosis, *C* crescent

## Discussion

The COVID-19 pandemic, caused by SARS-CoV-2, has had a significant impact on the global economy and public health [8]. Apart from the expected pulmonary involvement in SARS-CoV-2, other organ impacts have also been reported, including kidney involvement in patients with COVID-19 [9, 10]. SARS-CoV-2 infection may lead to various types of kidney damage [11, 12]. Although IgAN has been reported in association with SARS-CoV-2, these reports are limited to individual cases [13, 14]. To the best of our knowledge, this is the first study to compare clinical and pathological characteristics of IgAN between post-SARS-CoV-2 infection group and pre-SARS-CoV-2 infection group.

Starting from December 7, 2022, the relaxation of control measures in China, including the lifting of regional mass testing restrictions and the implementation of home isolation or quarantine, contributed to an unprecedented surge of the Omicron variant. As a result, there was a significant increase in the prevalence rate of COVID-19 [15]. A total of 48 patients were diagnosed IgAN from December 8, 2022 to April 6, 2023 in our hospital. These patients constituted approximately 22.5% of the total patients who underwent renal biopsy during that time. We analyzed the incidence rate of IgAN during the same time period before the COVID-19 outbreak and it ranged between 21.2 and 38.0% in recent three years in our center. Ten patients were excluded from the study due to their ineligibility for inclusion. Among the 38 patients who contracted SARS-CoV-2, 24 had a documented history of nephropathy, 8 had an uncertain nephropathy history, and 6 did not have any prior history of nephropathy before being infected with COVID-19. After SARS-CoV-2 infection, 13.2% of the patients (5/38) presented with gross hematuria. It is well known that many viral and bacterial pathogens have the ability to trigger gross-hematuria and increased proteinuria. Therefore, we can conclude that, as expected, COVID-19 share with other pathogens the ability to exacerbate the clinical features of IgAN. In recent years, there have been several reports of patients with preexisting or newly diagnosed IgAN who are susceptible to gross hematuria after receiving the severe acute respiratory syndrome coronavirus 2 (SARS-CoV-2) vaccine [16]. We also observed that 13.2% of the patients (5/38) experienced an increase in proteinuria after contracting SARS-CoV-2. These findings suggest that SARS-CoV-2 infection may lead to gross hematuria or an exacerbation of proteinuria in a subset of patients with IgAN. It is important to monitor and assess proteinuria levels in individuals with IgAN who have been infected with SARS-CoV-2 to ensure appropriate management and treatment of kidney-related complications.

It is not yet clear whether the occurrence of COVID-19 affects the presentation of the IgAN. One case reports have described five cases of IgAN with gross hematuria that were confirmed by biopsy after SARS-CoV-2 infection [14]. In one case among the reported cases, the individual experienced gross hematuria within two days after the onset of fever. The hematuria lasted for a duration ranging from 1 to 6 days. Additionally, it is worth noting that during the episode of gross hematuria, one patient developed acute kidney injury (AKI). In our study, we found that the cohort of patients infected with SARS-CoV-2 exhibited advanced age, a higher prevalence of hypertension and lower levels of eGFR compared to those in the pre-SARS-CoV-2 infection group. Although there was no statistically significant difference in diabetes incidence between the two groups, the post-COVID-19 infection group had a higher prevalence of diabetes. As for pathological characters, the post-COVID-19 infection group exhibited a higher proportion of sclerotic glomeruli and glomerular ischemic sclerosis compared to the pre-COVID-19 infection group. Even though there was no statistically significant difference in the tubular atrophy/interstitial fibrosis (T1/2), the post-COVID-19 infection group had a higher proportion of T1/2 lesions. This finding suggested the older age in patients with SARS-Cov-2 is consistent with the evidence in the literature and alone can explain the clinical and histological characteristics found in the cohort of patients with covid-19 infection. Indeed, multiple studies have consistently reported the elderly population is known to be more vulnerable to severe illness and complications associated with COVID-19 [13]. Advanced age is recognized as a significant risk factor for developing severe symptoms, experiencing higher rates of hospitalization, requiring intensive care, and facing an increased risk of mortality from COVID-19. Regular monitoring of renal function and appropriate medical care are crucial for identifying and managing any declines in eGFR or related renal complications in elderly individuals who contract COVID-19. As for treatment decision, we found the treatment approach remained consistent regardless of the presence of COVID-19.

So far, the pathogenic mechanism underlying COVID-19-associated kidney damage remains incompletely understood. It has been suggested that elevated levels of a circulating form of abnormal glycosylation (Gd-IgA1) may contribute to the pathogenesis of IgAN [17, 18]. COVID-19 virus primarily affects the respiratory tract, leading to the stimulation of excessive production of IgA1, including Gd-IgA1 [16]. Gd-IgA1 has the potential to recognize specific structures on certain microorganisms and form circulating complexes with them, thereby facilitating antigenic recognition. Previous studies have demonstrated that potential underlying mechanisms of kidney involvement in COVID-19 may include direct kidney infection through ACE-2 receptors expressed in tubular cells and podocytes [19]. Additionally, an indirect mechanism may involve cytokine release syndrome observed in COVID-19 patients [20]. The cytokines released during COVID-19 infection, including IL-1, IL-6, and TNF, have the potential to stimulate the proliferation and maturation of IgA1-producing B cells, thereby contributing to the development and severity of IgAN [21, 22]. In this study, we found no evidence of an increased incidence rate of IgAN during COVID-19 infection. However, we did observe that SARS-CoV-2 infection might be associated with severe kidney damage in these patients. The high prevalence of glomerular sclerosis among our post-COVID-19 infection group suggests a chronic disease trajectory in this population. While a higher proportion of T1/2 lesions suggests that COVID-19 infection may induce tubulointerstitial lesions in the kidney in IgAN, further research is needed to establish a clear causal relationship between COVID-19 and tubulointerstitial damage in this context. In this study, we observed that while Covid-19 infection can exacerbate IgAN, it does not contribute to an increased incidence of IgAN. This suggests that the virus is not a causal factor in the development of the disease.

The present study has several limitations that should be acknowledged. Firstly, it was a retrospective cohort study, which may introduce inherent biases and limit the generalizability of the findings. The small sample sizes in the post-COVID-19 infection group further impact the statistical power and reliability of the results. Secondly, the underlying mechanisms linking COVID-19 infection and the onset as well as clinical manifestations of IgAN remain unclear, and further research is warranted to elucidate these mechanisms.

## Conclusion

IgA nephropathy patients who were infected with SARS-CoV-2 were generally older and experienced more severe kidney damage compared to those without SARS-CoV-2 infection. This indicates that the presence of the virus may exacerbate the underlying glomerular disease, leading to more pronounced renal abnormalities, particularly in elderly patients. Further research is necessary to better understand the mechanisms underlying the relationship between SARS-CoV-2 infection and IgAN.

## Data Availability

Raw data used during the current study are available from the corresponding author on reasonable request for non-commercial use.
